# Frontal lifting using a tissue expander in pachydermoperiostosis: A case report

**DOI:** 10.1002/ccr3.3391

**Published:** 2020-12-18

**Authors:** Daniel José Dias Cunha, Rui Manoel Rodrigues Pereira, Julia Leal Dantas Vasconcelos Nassar, Edilson Xavier de Oliveira Junior, Jorge Luis Matta Ramos

**Affiliations:** ^1^ Instituto de Medicina Integral Prof. Fernando Figueira Recife Brazil

## Abstract

Pachydermoperiostosis, a rare condition, is characterized by pachydermia, finger clubbing, and periostosis. We present an unusual treatment for frontal rhytids, for which we used a tissue expander that contributed to thinning of the skin and the depth of the rhytids prior to frontal lifting. The results were maintained after one year.

## Graphical abstract

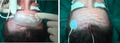

## 1 INTRODUCTION

Pachydermoperiostosis is the primary form of hypertrophic osteoarthropathy, which is characterized by pachydermia, finger clubbing, and periostosis. It should be differentiated from the secondary form of hypertrophic osteoarthropathy, which is more common and often associated with pulmonary bronchiectasis, bronchogenic carcinoma, congenital heart disease, and malignant neoplasms of the thyroid and gastrointestinal tracts.^1^ It was first described in 1868 by Friedrich, who reported two brothers with what he termed hyperostosis of the entire skeleton. In 1935, three dermatologists—Touraine, Solente, and Golé (for whom the syndrome was named afterward)—distinguished its three forms: complete form (with periostosis, pachydermia, and finger clubbing), incomplete form (without pachydermia), and *forme fruste* (pachydermia with minimal skeletal changes).^2^, ^3^ Its pathogenesis is not completely elucidated but is related to an increase in the secretion of prostaglandin E2. Regardless of the subtype, all patients present with acropathy; 20%–40% of patients present with arthralgia (associated or not with arthritis), mainly in the knees, hips, and hands, and joint effusion may also be present.^4^, ^5^ Cutaneous manifestations include thickening of the skin, mainly of the face and scalp, with prominent wrinkles in the form of *cutis verticis gyrata.* Edema and palpebral ptosis may be present due to the proliferation of dermal fibroblasts.^4^

Symptoms usually begin at puberty and tend to stabilize with time. Pachydermoperiostosis predominantly occurs in male individuals at a ratio of 9:1, with a familial inheritance pattern, and both autosomal dominant inheritance and autosomal recessive inheritance have been described.^4^, ^6^ Various treatments, such as rhytidoplasty, rhytidectomy, and botulinum toxin injections, have been described in the literature.^7^, ^8^, ^9^ Two cases involving frontal region treatment associated with the use of tissue expanders prior to frontal rhytidoplasty have also been reported.^9^, ^10^

A search was performed of the PubMed and Embase databases using the following terminology: pachydermoperiostosis, “Touraine Solente and Golé Syndrome,” treatment, surgical treatment, frontal lifting, subperiosteal lifting, tissue expander, and combinations of these. We aimed to report an unusual surgical treatment, the third case in the literature to the best of our knowledge, involving the frontal region in a patient with pachydermoperiostosis using a tissue expander before rhytidoplasty to thin the skin and decrease the prominent wrinkles and folds that are characteristic of the disease.

## 2 CASE REPORT

**Figure 1:**
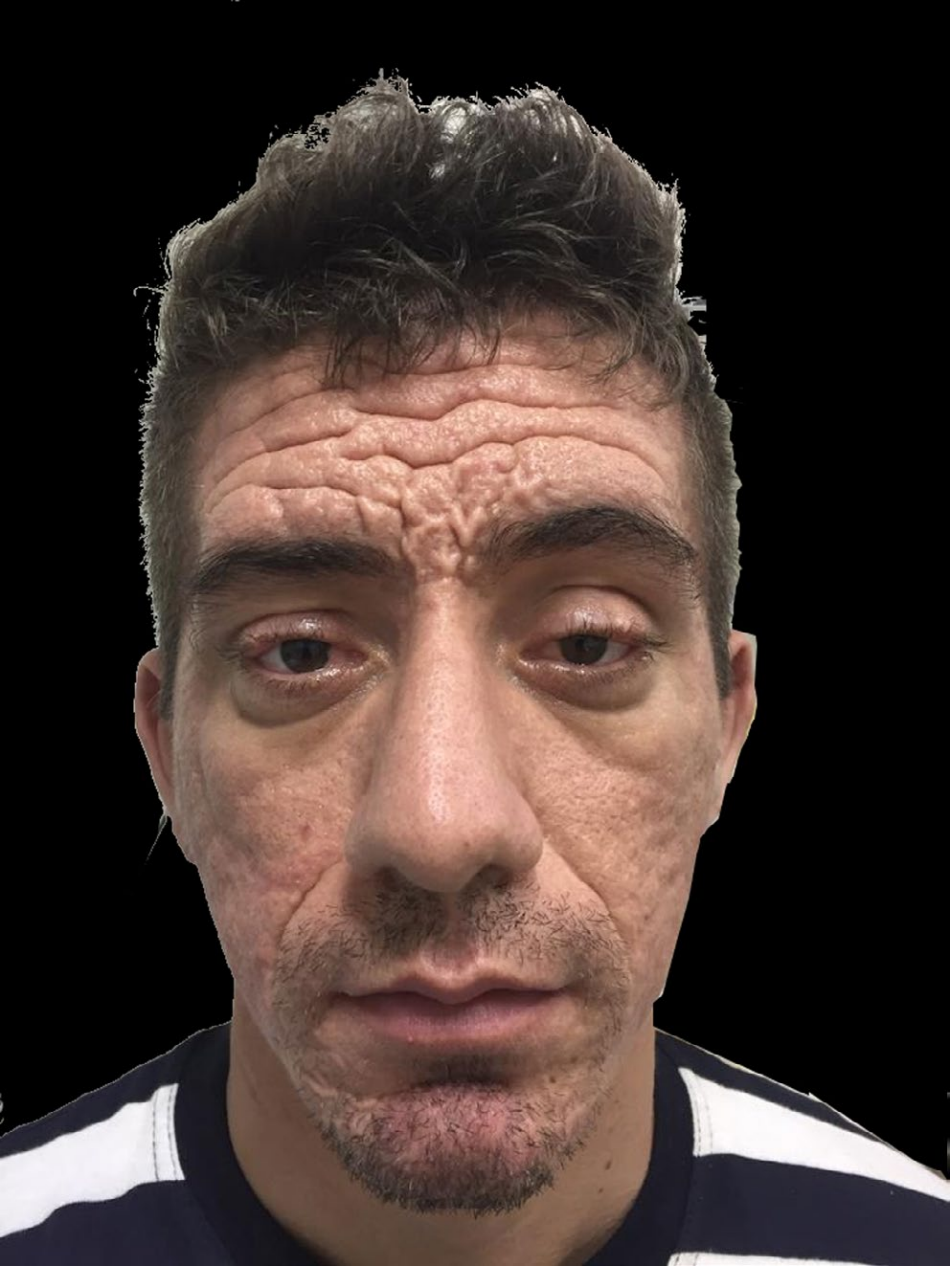
A 25‐year‐old patient with pachydermoperiostosis

**Figure 2:**
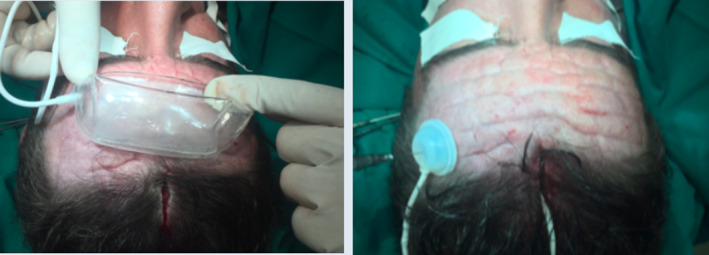
Patient who underwent placement of a tissue expander on the forehead in a submuscular position

A 25‐year‐old hypertensive man presented with a history of skin thickening mainly on the face and scalp from the age of 14 years associated with prominent skin wrinkles and folds (Figure 1). He also presented with joint edema in the hands and knees and finger clubbing. His main complaint was premature aging of his face, specifically the wrinkles in the frontal region. He had previously received botulinum toxin injections with unsatisfactory results. Preoperative examinations did not reveal any changes. Placement of a rectangular tissue expander was proposed to thin the skin and reduce the depths of the skin wrinkles and folds that are characteristic of the syndrome (Figure 2).

**Figure 3:**
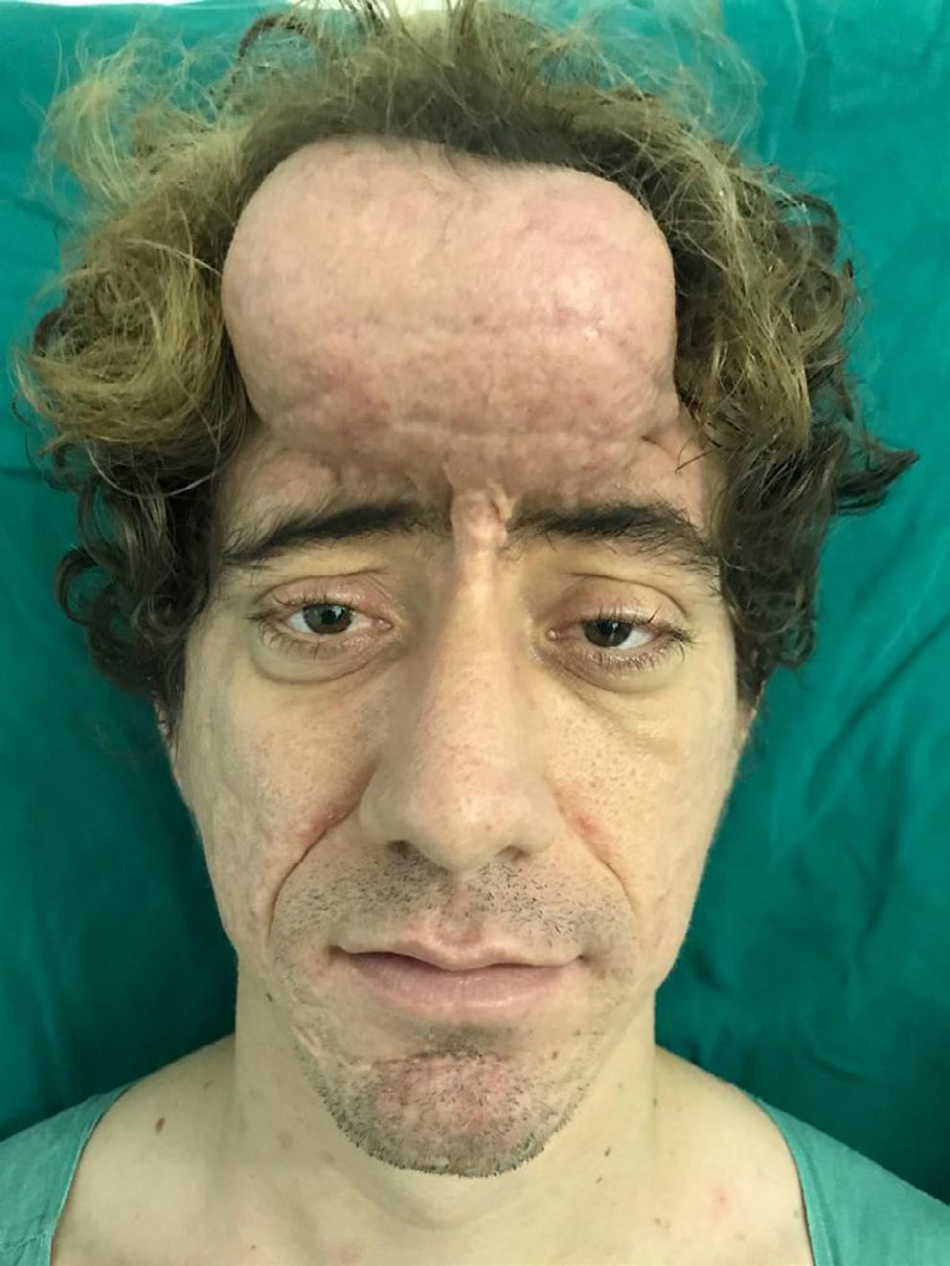
Slow and gradual expansion was performed until 200 mL was filled

Subperiosteal lifting was chosen, as the disease pathophysiologically manifests with thickened periosteum. In an attempt to thin the skin and decrease the prominent wrinkles and folds that are characteristic of the disease, as well as achieve greater and improved lengthening of the forehead skin over time compared with the punctual lengthening achieved via surgery, a rectangular expander measuring 8.5 × 5 cm with a 200 mL volume was initially placed on the forehead in a submuscular position. Slow and gradual expansion was then performed until the 200 mL volume was filled (Figure 3). Six months after the placement of the expander, the patient underwent a subperiosteal frontal lift, wherein a precapillary incision was made, the expander and its capsule were removed, and multiple incisions were made in the forehead flap to improve stretching. Resection of the thickened periosteum, superior flap traction, and removal of the excess skin were also performed, maintaining the previous capillary line.

## 3 OUTCOME

**Figure 4:**
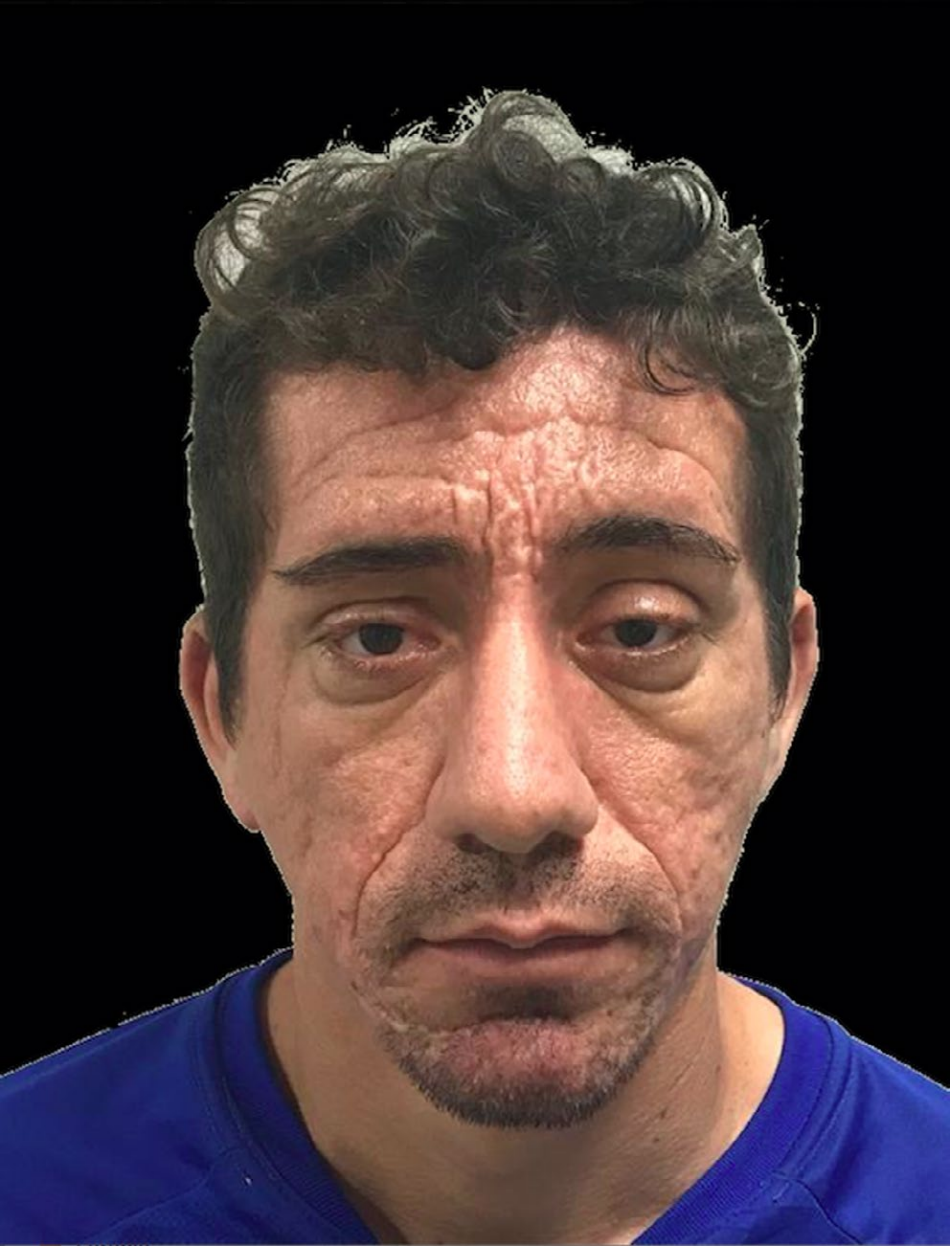
Postoperative result 1 y after the frontal lift

The patient presented significant improvements in the skin folds and wrinkles of the forehead, with this result being maintained 1 year after the surgical treatment (Figure 4). Slight scars were noted. The patient complained of hypoesthesia in the frontal region until the third month after the final surgery.

## 4 DISCUSSION

The classic methods of rejuvenation of the upper third of the face, such as botulinum toxin injections, are not effective in cases of pachydermoperiostosis as the primary cause of deep wrinkles is thick skin (mainly the dermis), rather than the repetitive use of the frontal and corrugator muscles. Rhytidoplasty with subperiosteal detachment of the upper third of the face is effective for the treatment of deep wrinkles, as it treats its cause, that is, thickened periosteum. The use of a tissue expander contributes to thinning of the skin and reductions in the depths of rhytids.

The syndrome described herein is rare and presents with phenotypic variability. Reports of new cases and treatments of pachydermoperiostosis (Touraine‐Solente‐Golé syndrome) are important to provide new individualized treatments according to the deformities presented.

## CONFLICT OF INTEREST

The authors declare no commercial associations or financial relationships that might create a conflict of interest.

## AUTHOR CONTRIBUTION

DJDC: participated in surgery, patient management, and drafting the manuscript. RMRP: participated in surgery, patient management, and drafting the manuscript. JLDVN: participated in surgery, patient management, and drafting the manuscript. EXOJ: participated in surgery, patient management, and drafting the manuscript. JLMR: participated in surgery, patient management, and drafting the manuscript.

## ETHICAL APPROVAL

This study was approved and complied with the World Medical Association Declaration of Helsinki on Ethical Principles for Research Involving Human Participants. Written free and informed consent was taken.

## References

[1] Vogel A , Goldfischer S . Pachydermoperiostosis: Primary or idiopathic hypertrophic osteoarthropathy. Am J Med. 1962;33:166‐187. 13926461 10.1016/0002-9343(62)90016-5

[2] Friedrich N . Hyperostose des gesammten skelettes. Virchows Arch Pathol Anat. 1868;43:83‐87.

[3] Touraine A , Solente G , Gole L . Un syndrome osteodermopathique: la pachydermie plicaturee avec pachyperiostose des extremites. Presse Med. 1935;43:1820e1824.

[4] Kumar S , Sidhu S , Mahajan BB . Touraine‐Soulente‐Golé syndrome: a rare case report and review of the literature. Ann Dermatol. 2013;25:352‐355. 24003280 10.5021/ad.2013.25.3.352 PMC3756202

[5] Zhang Z , Zhang C , Zhang Z . Primary hypertrophic osteoarthropathy: an update. Front Med. 2013;7:60‐64. 23345113 10.1007/s11684-013-0246-6

[6] Friedhofer H , Salles AG , Gemperli R , Ferreira MC . Correction of eyelid anomalies in pachydermoperiostosis. Ophthal Plast Reconstr Surg. 1999;15:137. 10.1097/00002341-199903000-00014 10189644

[7] Verjee LN , Greig AV , Kirkpatrick WN . Craniofacial strategies for the management of pachydermoperiostosis–a case report and review of the literature. J Plast Reconstr Aesthet Surg. 2009;62:e511‐e513. 19201670 10.1016/j.bjps.2008.08.036

[8] Salah BI , Husari KI , Hassouneh A , Al‐Ali Z , Rawashdeh B . Complete primary pachydermoperiostosis: a case report from Jordan and review of literature. Clin Case Rep. 2019;7:346‐352. 30847204 10.1002/ccr3.1971 PMC6389490

[9] Taichao D , Fuling L , Hengguang Z . Comprehensive surgical strategies for the management of pachydermoperiostosis. Facial Plast Surg. 2018;34:330‐334. 29763941 10.1055/s-0038-1653992

[10] Liu CY , Zhang YF . Images in clinical medicine. Pachydermoperiostosis. N Engl J Med. 2014;370:1930. 24827037 10.1056/NEJMicm1309107

